# The effect of family support on junior high school students' engagement in physical education—A moderated chain mediation model

**DOI:** 10.3389/fpsyg.2025.1606642

**Published:** 2025-08-04

**Authors:** Xiaoyu Wang, Yong Jiang

**Affiliations:** College of Physical Education, Liaoning Normal University, Dalian, China

**Keywords:** family support, engagement in physical education, academic self-concept, achievement goal orientation, academic burnout, exercise psychology, a moderated chain mediation model

## Abstract

**Introduction:**

Engagement in physical education constitutes an interactive process integrating experiential psychology and behavior, which positively contributes to sustaining adolescents‘ long-term physical activity adherence. This study explored the effects of family support on junior high school students' engagement in physical education, along with the mediating roles of academic self-concept and achievement goal orientation, and the moderating role of academic burnout within this mediation pathway.

**Methods:**

The study used the Family Support Scale, Engagement in Physical Education Scale, Academic Self-Concept Scale, Achievement Goal Orientation Scale, and Academic Burnout Scale to administer questionnaires to 1,059 junior high school students. Statistical analysis was performed on the processed data using SPSS 26.0. Mediation and moderation effects were examined respectively using Process 3.4 and Model 91 in the PROCESS macro, with the mediation effects further verified using AMOS 28.0.

**Results:**

(1) Family support significantly and positively predicts junior high school students' level of engagement in physical education; (2) Academic self-concept and achievement goal orientation serve not only as simple mediators in the influence of family support on engagement in physical education, but also transmit this influence through a chain: family support → academic self-concept → achievement goal orientation → engagement in physical education. (3) Academic burnout moderates between academic self-concept and achievement goal orientation.

## 1 Introduction

Engagement in education is characterized as a positive and fulfilling mental state associated with learning, representing an involved process encompassing behavior, cognition, and emotion (Skinner et al., 2009). The introduction of the concept of “engagement” into the domain of physical education constitutes a more proactive endeavor. While sharing the common emphasis on behavioral participation and the expression of willingness found in general engagement, engagement in physical education places stronger emphasis on the individual's proactivity, self-motivation, and self-discipline (Xiangning et al., 2021). Bevans et al. defined engagement in physical education as the individual's active exertion to acquire knowledge and skills, manifesting concretely as a preference for physical activities and an active participation process in physical education (Bevans et al., 2010). Engagement in physical education represents an experiential and behavioral interplay, holding positive implications for the sustained participation of individuals in physical activity (Miao et al., 2024). Individuals exhibiting lower levels of engagement in physical education demonstrate insufficient effort and attentional deficits, manifesting as more frequent evasive behaviors (Curran and Standage, 2017); concurrently, lower emotional engagement fosters negative affectivity, consequently leading to diminished study interest and motivation (Van den Berghe et al., 2015). Due to the open nature of the physical activity participation environment, the interplay between external environmental factors and individual psychological factors can also induce variations in an individual's level of engagement in physical education, ultimately impacting study outcomes (Kehong et al., 2021). Engagement in physical education manifests more prominently as an autonomous nature of the individual, and related discussions have predominantly focused on examining the effect of motivational variables on it. Solely incorporating a single theoretical variable into the consideration of antecedent factors for engagement in physical education results in limited explanatory power (Mingkai et al., 2021). By considering the environment and process of physical education study, this study integrates the relationship of different latent variables and deeply explores the antecedent elements of engagement in physical education, which contributes to clarifying and constructing the promotion mechanism of junior high school students' engagement in physical education.

## 2 Research hypothesis

### 2.1 Prediction of the effect of family support and junior high school students' engagement in physical education

According to Social Cognitive Theory proposed by American psychologist Albert Bandura (Alruwaie et al., 2021), human behavior emerges from the triadic reciprocal interaction among personal cognition, behavior, and the environment. Both internal cognitive modifications and external environmental alterations can influence an individual's behavioral change. The environment promotes the internalization of extrinsic motivation by supporting the fulfillment of individuals' basic psychological needs, thereby eliciting autonomous motivation, which ultimately impacts behavioral outcomes, such as engagement in physical education (Ryan and Deci, 2000). Research by Xin and Jiaxin (2013) demonstrated that parental behavioral encouragement, provision of equipment, and co-participation within the family environment significantly and positively predicted adolescent physical activity. Similarly, findings by Zhang Danqing et al. indicated that parental support, co-participation, and parental modeling are positively associated with adolescent physical activity levels (Danqing et al., 2019). In a systematic review examining parental influences on adolescent physical activity, Edwardson and Gorely established that parents positively influenced their children's physical activity via direct involvement, role modeling, encouragement, and provision of transportation (Edwardson and Gorely, 2010). Research by Pugliese et al. demonstrated that parental modeling, encouragement, and instrumental support were significantly associated with adolescent physical activity (Pugliese and Tinsley, 2007). Based on the above analysis, the present study proposes Hypothesis H1: Family support significantly predicts junior high school students' level of engagement in physical education.

### 2.2 Prediction of the mediating effect of academic self-concept in junior high school students

Academic self-concept refers to “an individual's relatively stable cognitive and affective assessment framework regarding their academic development within educational contexts” (Cheng et al., 2011). Within the theoretical framework of developmental systems theory, the family subsystem constitutes a critical interpersonal component of the social system influencing individual development (Mcmahon and Patton, 2018). It shapes offspring's academic self-concept through the provision of informational, emotional, and financial support, as well as by fostering culturally congruent behavioral expectations (Fouad et al., 2010). Research by Bryant et al. demonstrated that parenting styles exhibited significant correlations with the academic development of children and adolescents (Bryant et al., 2006). Specifically, positive parenting practices serve as positive predictors of adolescents' career exploration behaviors and attitudes (Kejia et al., 2016). Bandura's Social Cognitive Theory posits that intrinsic cognitive transformations within individuals influence their behavioral modifications (Alruwaie et al., 2011). Furthermore, findings by Connell et al. revealed that academic self-concept, functioning as a motivational system, exerted a substantial influence on students' academic engagement and study outcomes (Connell et al., 1994). Synthesizing the evidence, academic self-concept constitutes a latent predictor linking familial factors with academic engagement (Bakadorova and Raufelder, 2017; Basharpoor et al., 2022). Consequently, the present study proposes Hypothesis H2: Academic self-concept mediates the relationship between family support and junior high school students' engagement in physical education.

### 2.3 Prediction of mediating effects of achievement goal orientation in junior high school students

Achievement goal orientation refers to an individual's purpose or rationale for pursuing accomplishment in evaluative tasks (Elliot et al., 2011; Vandewalle, 1997), constituting a critical intrinsic motivational factor. Pintrich categorized this construct into mastery-approach goals, mastery-avoidance goals, performance-approach goals, and performance-avoidance goals (Pintrich, 2000b). The familial environment exerts a profound influence on students' achievement goal orientations, whereby positive and adaptive parenting practices enhance children's levels of achievement motivation (Yuntao and Hong, 2009). Research by Song Bing et al. demonstrated significant associations between maternal emotional involvement and both the formation of children's achievement goal orientation and their academic performance (Bing, 2010). Corroborating these findings, Jin Dan's research concluded that higher levels of nurturant understanding demonstrated by parents during child-rearing predicted a greater propensity for children to develop mastery-approach goal (Dan, 2011). Further empirical support came from Huang Shuang et al. whose findings indicate that when parents facilitated the internalization of attitudes, expectations, and affective states as personal values during educational processes, this subsequently fostered enhancement in achievement goal orientation (Shuang and Liyan, 2014). Human behavior emerges from the triadic reciprocal causation among personal cognition, behavior, and the environment (Alruwaie et al., 2011). As an intrinsic motivational construct, achievement goal orientation exerts direct effects on modifications in individual behavioral engagements (Vandewalle, 1997). Empirical research by He Liming on adolescents revealed that mastery-approach, performance-avoidance, and mastery-avoidance goals constituted significant predictors of study engagement levels (Liming, 2014). Corroborating evidence from Zhou Fang et al. further established that achievement goal orientation positively predicted adolescents' academic engagement (Fang, 2015). Synthesizing this theoretical and empirical foundation, the present study proposes Hypothesis H3: Achievement goal orientation mediates the relationship between family support and junior high school students' engagement in physical education.

### 2.4 Prediction of chain mediation of academic self-concept and achievement goal orientation

Self-Determination Theory posits that individuals are inherently proactive organisms with evolutionary tendencies toward intrinsic motivation and natural developmental progression through integrative processes. However, this development is contingent upon social-contextual conditions (Deci and Ryan, 2012). Crucially, the enhancement or diminishment of intrinsic motivation is determined by whether the social environment thwarts or supports the satisfaction of fundamental psychological needs for competence and autonomy (Chunmei, 2023). As the primary proximal context encountered earliest in development, the family system exerts sustained influence when parents demonstrate warmth and attunement while providing cognitively stimulating activities. Such family educational practices facilitate the long-term development of cognitive and physical skills, experiential study, and the internalization of success or failure attributions (Conger et al., 2007). Academic self-concept is subject to modulation by students' prior achievement outcomes (Zimmermann et al., 2018). According to Achievement Goal Theory, students endorsing divergent goal orientations operationalize “success” and “failure” through distinct evaluative frameworks (Elliot and Harackiewicz, 1996). Empirical evidence confirmed that heterogeneous achievement goal orientations exert differential effects on the construction of academic self-concept across student populations, subsequently manifesting as variations in engagement levels (Zhiguo et al., 2023). Synthesizing this theoretical and empirical foundation, the present study proposes Hypothesis H4: Academic self-concept and achievement goal orientation function as chain mediators in the relationship between family support and engagement in physical education among junior high school students.

### 2.5 Prediction of moderating effects of academic burnout in junior high school students

Academic burnout constitutes a chronic, negatively valenced psychological phenomenon primarily observed in student populations (Yan et al., 2007), characterized by three core dimensions: emotional exhaustion, academic disengagement, and reduced sense of personal accomplishment (Maslach et al., 2001). Empirical research by Reschly et al. demonstrated that this condition leads to multiple adverse educational consequences, including diminished academic motivation and impaired school adaptation (Reschly et al., 2008). Social Cognitive Theory posits that human behavior is mediated substantially by self-referential thought (Bandura, 1996). Academic self-concept embodies students' self-perception of their scholastic capabilities, constituting a relatively stable self-appraisal of academic competence (Cheng et al., 2011). As a primary intrapersonal determinant, self-concept significantly influences burnout development (Friedman and Farber, 1992). Empirical evidence consistently has demonstrated that elevated self-concept correlates with lower burnout levels—a finding corroborated by Villa and Calvete et al. who documented an inverse correlation between self-concept and burnout (Villa and Calvete, 2001). Furthermore, research by Schaufeli et al. revealed that adolescents' emotional exhaustion and depersonalization stemming from curricular pressures, academic overload, or psychological stressors during study processes are associated with declines in achievement goal orientation (Schaufeli et al., 2002). The preceding analysis suggests that academic burnout may potentially compromise inhibit the relationship academic self-concept into achievement goal orientation. Synthesizing this theoretical and empirical foundation, the present study proposes Hypothesis H5: Academic burnout moderates the relationship between academic self-concept and achievement goal orientation in the chain mediation of “family support → academic self-concept → achievement goal orientation → engagement in physical education”.

**Figure 1:**
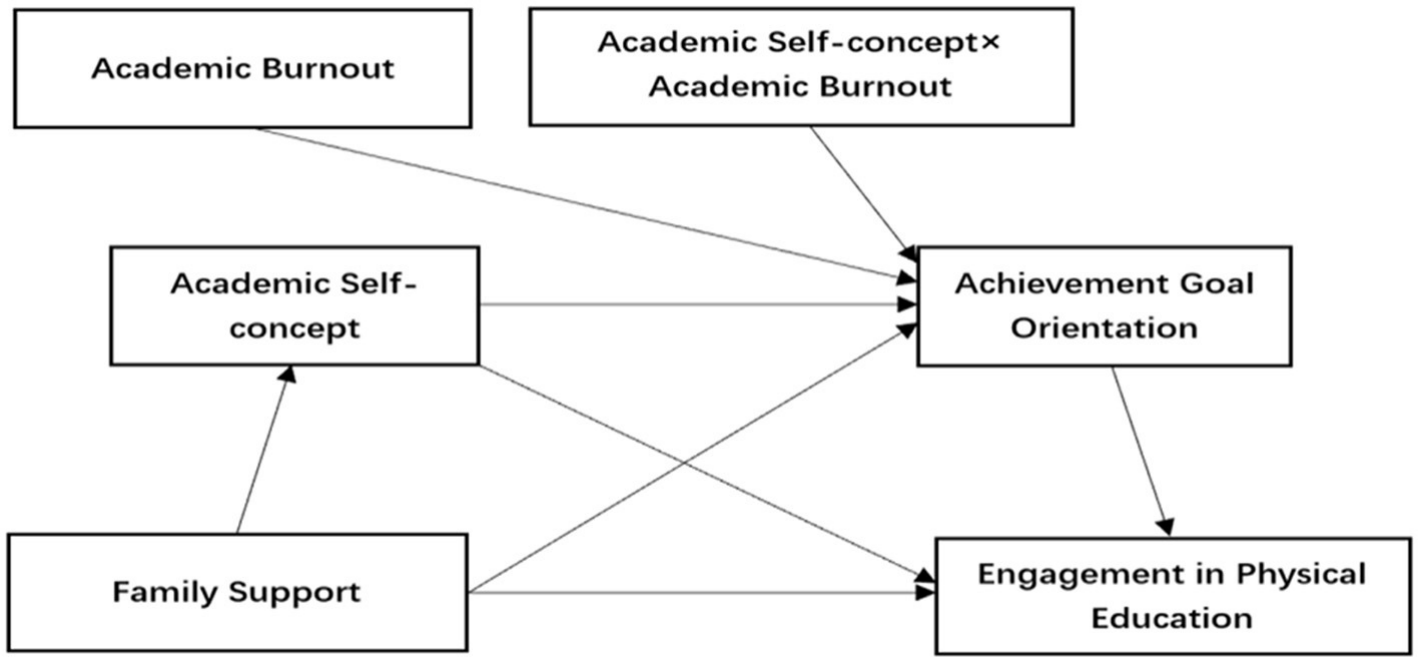
Diagram of the hypothesized model.

As a result, the research hypothesis model was constructed as shown in Figure 1:

## 3 Research objectives and methods

### 3.1 Research objectives

The object of this paper is to investigate the effects of family support on junior high school students' engagement in physical education and the mediating role of academic self-concept and achievement goal orientation, as well as the moderating effect of academic burnout between academic self-concept and achievement goal orientation. Sample size was predicted using G^*^Power 3.1 software with α set to 0.05, statistical test power (1-β) set to 0.99, and effect size set to a medium value (*f*^2^ = 0.15). The results indicated that a sample size of 261 was required. Employing a stratified cluster convenience sampling design, data collection took place in October 2024 across 12 junior high schools in Dalian, Liaoning Province. The sampling frame comprised three strata corresponding to grade levels (Grades 7–9). Administrative classes served as primary sampling units, with approximately 30 students per class selected through convenience sampling, generating an initial cohort of 1,080 participants. Exclusion of 21 invalid questionnaires due to factors such as short completion time and patterned responses, the effective sample size of this study was 1059 (age: M ± SD = 14.28 ± 1.12), and the sample validity rate was 98.06%. Details of the sampling procedure are presented in Table 1, and the final sample size met the criteria.

**Table 1:** Demographic characteristics of survey participants.

**Variant**	**Classification**	**Numbers**	**Percentage %**
Gender	Male	582	54.96
	Female	477	45.04
Grade	1	353	33.33
	2	350	33.05
	3	356	33.62
Birthplace	Urban	614	57.98
	Rural	445	42.02
Whether an only child	Yes	646	60.97
	No	413	39.03
Total		1,059	100

### 3.2 Research instruments

#### 3.2.1 The family support scale

This study measured family support, as an indicator of the physical and behavioral environment of family sports, using the Family Support Scale published by Peijie (2016) in the Report on the Development of Sports and Fitness for Children and Youth in China. The Family Support Scale comprises both subjective and objective support dimensions, totaling 10 items. Items were scored on a 5-point Likert scale (1 to 5), with higher scores indicating higher levels of family support. The overall Cronbach's α for the scale was 0.821.

#### 3.2.2 The engagement in physical education scale

The scale used in this study was adapted by Baogen et al. (2020) for the physical education context and was revised based on the Chinese version of the Student Engagement Scale (UWES-S) revised by Laitan et al. (2008). The revised scale measured the three dimensions of students' engagement in physical education: vigor, dedication and concentration, with a total of 17 items. Items were scored on a 5-point Likert scale (1 to 5), with higher scores indicating higher levels of engagement in physical education. The overall Cronbach's α for the scale was 0.97.

#### 3.2.3 The academic self-concept scale

This study used the 20-item Academic Self-Concept Scale developed by Cheng et al. (2011), which measures four dimensions: academic affective experience, academic achievement value, academic ability perception, and academic behavioral self-control. Items were scored on a 5-point Likert scale (1 to 5), with higher scores indicating higher levels of academic self-concept. The overall Cronbach's α for the scale was 0.93.

#### 3.2.4 The achievement goal orientation scale

This study used the Achievement Goal Orientation Scale developed by Huijun and Dejun (2003). This scale was based on Pintrich's (2000a) four-dimensional Achievement Goal Orientation Classification and adapted for the Chinese context. The scale consists of 29 items measuring four dimensions: mastery-approach goals, mastery-avoidance goals, performance-approach goals, and performance-avoidance goals. Items were scored on a 5-point Likert scale (1 to 5), with higher scores indicating higher levels of achievement goal orientation. The overall Cronbach's α for the scale was 0.831.

#### 3.2.5 The academic burnout scale

This study used the academic burnout questionnaire developed by Yan et al. (2007). The scale measures three dimensions: emotional exhaustion, cynicism, and reduced personal accomplishment, comprising 16 items. Items were scored on a 5-point Likert scale (1 to 5), with higher scores indicating higher levels of academic burnout. The overall Cronbach's α for the scale was 0.73.

### 3.3 Research methods

Statistical analysis was performed on the processed data using SPSS 26.0. Mediation and moderation effects were examined respectively using Process 3.4 and Model 91 in the PROCESS macro, with the mediation effects further verified using AMOS 28.0. These analytical procedures meet academic publishing requirements.

## 4 Results and analysis

### 4.1 Common method bias assessment

Due to factors such as measurement environment, questionnaire instructions, and contextual influences, survey data may exhibit common method bias. To assess common method bias, we performed Harman's single-factor test on all variables. Using SPSS 26.0, we conducted an unrotated principal component analysis on these variables. The results extracted 18 components with eigenvalues exceeding 1. The first component explained 28.60% of the variance, below the critical threshold of 40%. This suggests the absence of significant common method bias in this study's data.

### 4.2 Correlation analysis of variables

Bivariate Pearson correlation analysis revealed statistically significant associations among family support, engagement in physical education, academic self-concept, achievement goal orientation, and academic burnout, as shown in Table 2, the results indicate: family support showed positive correlations with engagement in physical education, Academic self-concept, and achievement goal orientation; Both academic self-concept and achievement goal orientation were positively correlated with engagement in physical education; Academic burnout was negatively correlated with academic self-concept and achievement goal orientation.

**Table 2:** Bivariate Pearson correlation analysis of the variables.

**Variant**	**1**	**2**	**3**	**4**	**5**
Family support	1				
Engagement in physical education	0.483^**^	1			
Academic self-concept	0.376^**^	0.415^**^	1		
Achievement goal orientation	0.433^**^	0.512^**^	0.468^**^	1	
Academic burnout			−0.278^**^	−0.362 ^**^	1

^**^p < 0.01.

Variant 1 indicates “Family Support”; Variant 2 indicates “Engagement in Physical Education”; Variant 3 indicates “Academic Self-concept”; Variant 4 indicates “Achievement Goal Orientation”; Variant 5 indicates “Academic burnout.”

Three separate forced-entry regression analysis were conducted with engagement in physical education as the dependent variable, using family support, academic self-concept, and achievement goal orientation as independent variables respectively. As shown in Table 3, the results indicate: Family support significantly predicted engagement (β = 0.698, *p* < 0.001), explaining 55.2% of the variance; Academic self-concept significantly predicted engagement (β = 0.642, *p* < 0.001), accounting for 44.6% of the variance; Achievement goal orientation significantly predicted engagement (β = 0.634, *p* < 0.001), accounting for 43.6% of the variance. According to self-determination theory, environmental factors facilitate behavioral change by supporting motivation internalization through basic psychological need satisfaction. As an external environmental factor, family support functions as the initial predictor in the chain mediation model. Its effects propagate through multiple mediating pathways, demonstrating stronger explanatory power than the individual contributions of academic self-concept and achievement goal orientation.

**Table 3:** Independent regression analyses of family support, academic self-concept, and achievement goal orientation on physical education engagement.

**Variant**	**Engagement in physical education**					
	***B***	***SE***	β	***T***	***F***	$R_{adj}^{2}$
Constant	2.937	0.056				
Family support	0.496	0.022	0.698^**^	24.372	561.18	0.552
Constant	1.786	0.112				
Academic self-concept	0.578	0.032	0.642^**^	21.264	434.52	0.446
Constant	1.636	0.119				
Achievement goal orientation	0.638	0.034	0.634^**^	21.196	406.48	0.436

^**^p < 0.01.

$R_{adj}^{2}$ is adjusted R^2^.

### 4.3 Mediation effect analysis

Using family support as the independent variable, engagement in physical education as the dependent variable, and academic self-concept and achievement goal orientation as the mediator variables, the chain mediation effect of academic self-concept and achievement goal orientation between family support and engagement in physical education was tested using SPSS 26.0 and PROCESS 3.4 plug-ins controlling for demographic variables such as gender, and the results are shown in Table 4. Procedures were as follows: in the first step, engagement in physical education was specified as the dependent variable, and gender, grade level, place of origin, and only-child status were included as control variables to account for their potential influence. In the second step, family support was added to the model to examine its total effect on engagement in physical education after controlling for the covariates. Results indicated that family support significantly and positively predicted engagement in physical education (β = 0.596, *p* < 0.001), supporting research hypothesis H1. In the third step, academic self-concept and achievement goal orientation were sequentially added to the model to test their roles as mediating variables between family support and engagement in physical education and whether a chain mediation effect existed. Results showed that family support remained a significant positive predictor of engagement in physical education (β = 0.284, *p* < 0.001). Additionally, academic self-concept (β = 0.364, *p* < 0.001) and achievement goal orientation (β = 0.276, *p* < 0.001) also significantly and positively predicted engagement in physical education. Furthermore, analysis revealed significant associations among the mediating variables: family support significantly and positively predicted academic self-concept (β = 0.516, *p* < 0.001) and achievement goal orientation (β = 0.258, *p* < 0.001). Academic self-concept also significantly and positively predicted achievement goal orientation (β = 0.602, *p* < 0.001). Based on the above regression coefficients, a significant chain mediation effect of academic self-concept and achievement goal orientation was observed between family support and engagement in physical education, supporting research hypotheses H2 and H3. Structural equation modeling (SEM) analysis was performed using AMOS 26.0 to test the mediation model. The results indicated a good-fitting model: χ^2^/*df* = 2.732, CFI = 0.903, TLI = 0.933, RMSEA = 0.059, SRMR = 0.048.

**Table 4:** Mediated effects regression model of academic self-concept and achievement goal orientation.

**Variant**	**Engagement in physical education**		**Academic self-concept**		**Achievement goal orientation**		**Engagement in physical education**	
	β	***t***	β	***t***	β	***t***	β	***t***
Gender	0.039	1.315	0.152	4.112^***^	−0.034	−0.822	−0.024	−0.572
Grade	0.028	0.556	0.022	0.203	−0.045	−1.324	0.034	0.776
Birthplace	−0.034	−1.124	0.026	0.462	0.012	0.306	−0.052	−1.912
Whether an only child	−0.017	−0.466^*^	0.062	1.227	−0.032	−0.954	−0.046	−1.468
Family Support	0.596	18.126^***^	0.516	13.536^***^	0.258	6.556^***^	0.284	7.896^***^
Academic burnout			−0.294	−16.782^***^	−0.372	−19.316^***^		
Academic Self-concept					0.602	18.394^***^	0.364	8.824^***^
Interaction term					−0.246	−14.926^***^		
Achievement Goal Orientation							0.276	7.428^***^
R^2^	0.424		0.276		0.586		0.582	
ΔR^2^	0.356		0.238		0.572		0.578	
F	51.486^***^		31.261^***^		88.274^***^		82.682^***^	

^*^p < 0.05 ^***^p < 0.001.

The interaction term is the academic self-concept with academic burnout centred on the interaction term.

As shown in Table 5, Bootstrap analysis with 5,000 resamples was performed to test the significance of the mediating effects of academic self-concept and achievement goal orientation between family support and engagement in physical education and to estimate their bias-corrected confidence intervals. The results showed that the total effect of family support on engagement in physical education was 0.596, with a 95% bias-corrected bootstrap confidence interval not containing zero (LLCI = 0.534, ULCI = 0.652), indicating a significant total effect. Furthermore, the 95% confidence intervals for the specific mediating effects (reported in Table 5) did not contain zero, indicating that these mediating effects were also significant. The direct effect of family support on engagement in physical education was 0.284, with a 95% bias-corrected bootstrap confidence interval not containing zero (LLCI = 0.256, ULCI = 0.384), indicating a significant direct effect, accounting for approximately 47.67% of the total effect.

**Table 5:** Mediating effects of academic self-concept and achievement goal orientation.

**Effect**	**Path**	**Effect value**	**Standard error**	**LLCL**	**ULCL**	**Efficiency ratio**
Total effect		0.596	0.038	0.534	0.652	100%
Direct effect	Direct path	0.284	0.036	0.256	0.384	47.65%
Total indirect effect		0.312	0.038	0.226	0.358	52.35%
Indirect effect	Path 1	0.136	0.034	0.061	0.184	22.82%
	Path 2	0.082	0.026	0.012	0.147	13.76%
	Path 3	0.094	0.032	0.035	0.165	15.77%

Path 1 indicates family support → academic self-concept → engagement in physical education; Path 2 indicates family support → achievement goal orientation → engagement in physical education; Path 3 indicates family support → academic self-concept → achievement goal orientation → engagement in physical education.

The model indicates that family support predicts engagement in physical education and that academic self-concept and achievement goal orientation serve as mediators via three distinct pathways. The total indirect effect was 0.312, with a Bootstrap 95% confidence interval not containing zero (LLCI = 0.226, ULCI = 0.358), accounting for 52.35% of the total effect. Specifically, the first mediating pathway (family support → academic self-concept → engagement in physical education) had an indirect effect of 0.136, accounting for 22.82% of the total effect; The second mediating pathway (family support → achievement goal orientation → engagement in physical education) had an indirect effect of 0.082, accounting for 13.76% of the total effect; The third, chained mediating pathway (family support → academic self-concept → achievement goal orientation → engagement in physical education) had an indirect effect of 0.094, accounting for 15.77% of the total effect. These results support Hypothesis H4 regarding the chained mediation mechanism.

### 4.4 Moderation effect analysis

Following the testing procedure for moderation effects outlined by Zhonglin et al. (2002), all variables were standardized. Using Model 91 in the PROCESS macro (with 5,000 bootstrap samples), we constructed a moderated mediation model. The chain-mediated effect values and 95% Bootstrap confidence intervals for academic self-concept and achievement goal orientation between family support and junior high school students' engagement in physical education at different levels of academic burnout are shown in Table 6. The results showed that the chain-mediated effect tended to decrease with increasing levels of academic burnout, which validated hypothesis H5. Academic burnout moderates the relationship between academic self-concept and achievement goal orientation in this chain-mediated effect. When the level of academic burnout is high, academic self-concept does not significantly predict achievement goal orientation; when it is low, academic self-concept significantly and positively predicts achievement goal orientation. As the level of academic burnout increases, the predictive effect of academic self-concept on achievement goal orientation gradually weakens.

**Table 6:** Chain-mediated effects at different levels of academic burnout.

**Academic burnout**	**Effect value**	**Standard error**	**LLCL**	**ULCL**
M−1SD	0.386	0.008	0.328	0.441
M	0.312	0.006	0.226	0.358
M + 1SD	0.239	0.005	0.202	0.276

**Figure 2:**
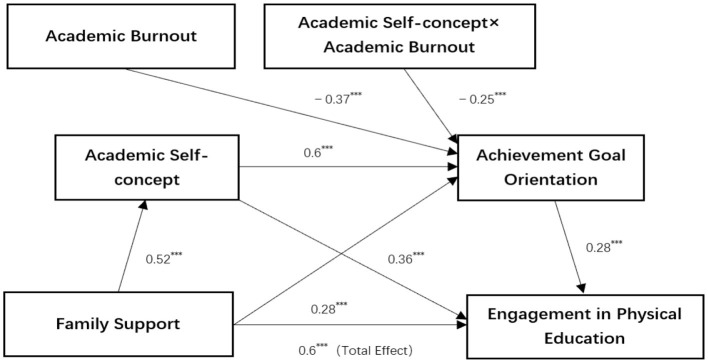
Moderated chain mediation effects modeling. ^**^p < 0.01.

Based on the aforementioned findings, the moderated chain mediation model of this study is illustrated in Figure 2.

## 5 Discussion

### 5.1 Family support positively predicts junior high school students' engagement in physical education

The results of the study showed that family support significantly and positively predicted junior high school students' engagement in physical education, thus verifying the validity of Hypothesis 1. Parental autonomy support for their children's participation in sport is a correctly oriented parenting style. It helps junior high school students express their true selves and pursue personal change, development, and growth through physical activity, ultimately leading to a higher level of engagement in physical education (Weijun, 2022; Mcdavid et al., 2012). Supportive physical education study environments are conducive to the transformation of autonomous motivation. In fostering supportive physical education study environments, parents can provide their children with appropriate autonomy and choice to satisfy their basic psychological needs. This motivates children to develop intrinsic motivation for study and to engage more autonomously in behaviors, cognitions, and emotions, resulting in more proactive and persistent engagement in physical education (Meng, 2020). At the same time, if families give their children better material support, including sports equipment, facilities, and a family atmosphere conducive to physical activity, it will help to improve children's perceptions of and attitudes toward physical activity, which can indirectly enhance children's engagement in physical education (Baolin and Lijuan, 2020; Lijuan and Danheng, 2020).

### 5.2 Academic self-concept mediates between family support and junior high school students' engagement in physical education

The study showed that academic self-concept partially mediates the relationship between family support and junior high school students' engagement in physical education (with a relative mediating effect = 22.82%). This indicates that family support not only directly affects students' engagement in physical education but also indirectly enhances it through improving their academic self-concept, thereby verifying Hypothesis 2. Self-Determination Theory postulates that an individual's developmental trajectory is contingent upon social-contextual conditions, with the attenuation or enhancement of intrinsic motivation determined by whether the social environment thwarts or supports the satisfaction of fundamental psychological needs for competence and autonomy (Li et al., 2019). Within families, parental warmth, positive affect, and provision of cognitively stimulating activities represent educational practices that facilitate the intergenerational transmission of parental resources. This transmission mechanism significantly enhances offspring's academic self-concept (Jiali, 2017). According to the socio-cultural self model, family support conditions and individual characteristics are interdependent, both indirectly influencing behavior through academic self-concept (Stephens et al., 2012). Students with stronger academic self-concept are more likely to participate actively in physical education activities, demonstrating higher cognitive engagement (Liem et al., 2008). Individuals with a high level of academic self-concept tend to have a more positive self-evaluation and greater confidence in their academic abilities, which fosters stronger motivation. As motivation serves as an intrinsic driver for study (Weili et al., 2022), this ultimately increases individuals' engagement in physical education.

### 5.3 Achievement goal orientation mediates between family support and junior high school students' engagement in physical education

The study showed that achievement goal orientation partially mediates the relationship between family support and junior high school students' engagement in physical education (with a relative mediating effect = 13.76%). This indicates that family support not only directly affects students' engagement in physical education but also indirectly enhances it through improving their achievement goal orientation, thereby verifying Hypothesis 3. Different achievement goal orientations lead to different behaviors, thereby resulting in different levels of engagement (Yong and Wei, 2023). Family support can shape students' achievement goal orientation. If family support focuses on 'encouraging attempts and tolerating failures', students will be more inclined to form 'mastery-approach goals', focusing more on enhancing their own skills and fulfilling their interests; on the contrary, if family support is more utilitarian, students may be prompted to form 'performance-approach goals' (Zaihua, 2022). Individuals with a high level of mastery-approach goals believe that they can change and grow their abilities through effort. Viewing their effort in learning activities as a means to grow their abilities, they tend to be more active and diligent in these activities, resulting in a higher level of engagement in physical education (Elliott and Dweck, 1988).

### 5.4 Academic self-concept and achievement goal orientation serve as chain mediators between family support and junior high school students' engagement in physical education

This study demonstrated the effects of family support, academic self-concept, and achievement goal orientation on junior high school students' engagement in physical education, with a total effect of β = 0.5963. The family support, academic self-concept, and achievement goal orientation each showed significant correlations with engagement in physical education. The direct effect of family support on engagement was β = 0.2841, while the total indirect effect was β = 0.3122. This indicates that the mediating effects accounted for a larger proportion of the total effect than the direct effect. Specifically, the third chained mediation path (family support → academic self-concept → achievement goal orientation → engagement in physical education) accounted for 15.77% of the total effect. This demonstrates the existence of the chain mediation effect, thereby verifying Hypothesis 4. This pathway empirically supports the core idea of social cognitive theory—that individual cognition (academic self-concept) drives behavior change (engagement in physical education) by influencing motivational processes (achievement goal orientation). Individual behavior is controlled by individual intentions, which in turn depend on the individual's attitudes toward the behavior, subjective norms and perceived behavioral control (Zhu and University B. S., 2020; Duan and Jiang, 2008). Engagement in physical education is a specific expression of individual cognition within the domain of physical education (Kaiming, 2021). Academic self-concept, defined as an individual's perception of their own academic ability, enables students to influence and regulate their achievement goal orientation through their self-knowledge and self-evaluation regarding learning (Seaton et al., 2014). During the study process, different achievement goal orientations affect the level of engagement by influencing task selection and task execution (Lifang, 2006). Therefore, a high academic self-concept activates an individual's achievement goal orientation, which in turn facilitates increased engagement in physical education.

### 5.5 Academic burnout moderates the relationship between academic self-concept and achievement goal orientation in the chain mediation model

The study showed that academic burnout moderated the relationship between academic self-concept and achievement goal orientation in the chain mediation model. Specifically, as academic burnout increased, the positive predictive effect of academic self-concept on achievement goal orientation weakened (Jones et al., 2012; McClun and Merrell, 1998), thus verifying Hypothesis 5. The core manifestation of academic burnout lies in the depletion of psychological resources, including emotion, cognition, and energy (Anming et al., 2022). When students experience emotional exhaustion and feelings of detachment, their willingness and ability to translate goals into practical action decrease significantly. Furthermore, these feelings of detachment may lead students to doubt or devalue the value of their study activities (Caiyu et al., 2023). Even if a student has a high academic self-concept, a high level of academic burnout can severely deplete both mental energy and intrinsic motivation necessary for pursuing goals (Anming et al., 2019). For students experiencing low academic burnout, high academic self-concept strongly predicts positive achievement goal orientations; however, for those with high academic burnout, even a non-low academic self-concept has greatly diminished efficacy in predicting positive achievement goal orientations (Zhuo et al., 2024).

## 6 Conclusions and outlook

### 6.1 Conclusions

This study proposes a moderated chain mediation model integrating research on the relationships among academic self-concept, achievement goal orientation, and engagement in physical education. This model facilitates a more comprehensive understanding of the internal mechanism through which family support influences junior high school students' engagement in physical education. First, significant correlations existed among family support, academic self-concept, achievement goal orientation, and engagement in physical education. Second, family support significantly and positively predicted junior high school students' engagement in physical education and represents an important factor influencing its development. Third, academic self-concept and achievement goal orientation serve not only as simple mediators in the influence of family support on engagement in physical education, but also transmit this influence through a chain: family support → academic self-concept → achievement goal orientation → engagement in physical education. This indirect (chain) mediation effect significantly contributes to promoting junior high school students' engagement in physical education. Fourth, academic burnout moderates the relationship between family support and engagement in physical education. Specifically, within the proposed chain mediation pathway (family support → academic self-concept → achievement goal orientation → engagement in physical education), academic burnout moderates the link between academic self-concept and achievement goal orientation. As academic burnout increases, the positive association between academic self-concept and achievement goal orientation decreases.

### 6.2 Outlook

Released in March 2021, the Chinese government's Outline of the Fourteenth Five-Year Plan for National Economic and Social Development and the Long-Range Objectives Through the Year 2035 proposes improving mechanisms for collaborative education among schools, families, and society. Consequently, home-school cooperation has become a prominent topic in China's education policy, practice, and academic research. The present study showed that family support significantly and positively influenced junior high school students' engagement in physical education. Furthermore, it revealed that academic self-concept and achievement goal orientation served not only as simple mediators in this relationship, but also exhibited a significant chain mediation effect. The study revealed that family support plays a unique role in promoting junior high school students' engagement in physical education, with academic self-concept and achievement goal orientation playing significant mediating roles in this process. Accordingly, the study advocates breaking the traditional fragmented approach where schools, families, and communities operate in isolation, eliminating the gaps between them, and establishing a collaborative mechanism.

As a cross-sectional study, this research has limitations. Specifically, focusing solely on junior high school students limits its ability to infer causality. Future studies should expand the population scope and sample size, and utilize longitudinal designs to explore the intrinsic causal relationships among the variables.

## Data Availability

The raw data supporting the conclusions of this article will be made available by the authors, without undue reservation.
